# Do Generic Skills Achieved During Undergraduate Dental Education Meet the Need in Clinical Practice?

**DOI:** 10.1111/eje.70037

**Published:** 2025-08-14

**Authors:** Ritva Näpänkangas, Olli‐Pekka Lappalainen, Hannaleena Jämsä, Marja‐Liisa Laitala, Jaakko Koivumäki, Terhi Karaharju‐Suvanto

**Affiliations:** ^1^ Research Unit of Population Health University of Oulu Oulu Finland; ^2^ Medical Research Center Oulu University Hospital and University of Oulu Oulu Finland; ^3^ Department of Oral and Maxillofacial Diseases, Faculty of Medicine University of Helsinki Helsinki Finland; ^4^ Helsinki University Hospital Oral Diseases Teaching and Treatment Unit Helsinki Finland; ^5^ Finnish Dental Association Helsinki Finland

**Keywords:** competence, dental education, generic skills, undergraduate education

## Abstract

**Objectives:**

To evaluate the importance of generic skills in clinical dental practice and how well these skills were achieved during undergraduate dental education.

**Materials and Methods:**

The data were based on a national online questionnaire survey called ‘Young Dentist’, which was hosted by the Finnish Dental Association. The questionnaire was sent via an email link in May 2021 to dentists who had graduated in Finland during 2018–2020. Altogether 221/505 recently graduated dentists (response rate 44%) answered the questionnaire. At the same time, the questionnaire was also sent to more experienced colleagues, who were selected by random sampling from the member register of the Finnish Dental Association (response rate 82/778, 14%).

**Results:**

Recently graduated dentists stated that patient encounter skills were the most important generic skill in clinical dental practice, and this skill was well achieved during undergraduate studies. Other important skills in clinical dental practice reported by young dentists were aseptic techniques and patient safety and risk management skills, which were also well or very well achieved during undergraduate studies. The opinions of recently graduated dentists and more experienced colleagues were quite similar.

**Conclusion:**

According to the opinions of the dentists, undergraduate dental education develops most generic competencies in clinical dental practice, but evolving practices, advancing technologies and health system challenges highlight the growing importance of diverse generic skills in the dental profession.

## 1 Introduction

The Association for Dental Education in Europe (ADEE) presented the competences for the graduating dentist in 2004, with an update approved in 2009 [1]. In 2017, the revised curriculum framework was published with four domains of learning outcomes: Professionalism; safe and effective clinical practice; patient‐centred care; and dentistry in society [2]. The uniform competencies in Europe published by ADEE aim to refine and harmonise the structure and quality of dental undergraduate educational programmes in Europe so as to also promote the mobility of dentists on a European level [2]. Cowpe et al. [1] defined that ‘competences are professional behaviours and skills required by a graduating dentist in order to respond to the full range of circumstances encountered in general professional practice’. Undergraduate dental education should offer competences for future dentists to work in all areas in the labour market, as members of working teams in the dental office and on society level. The professional skills must also meet the needs of different working sectors, taking into the account the fact that the special features of each working sector emphasise different skills.

Competence is very much a professional construct [1], and it is obvious that competence will be well achieved in skills that are common in dental practice not only by dental students but also by dentists. It is also evident that in all health care professions lifelong development of competences is an essential part of managing successfully the rapid evolution in medical information and practice [3]. The competence of graduating dentists during the undergraduate education has been evaluated in the literature [4, 5, 6]. The undergraduate dental students have been shown to be most competent in producing and maintaining accurate patient records, implementing sterilisation and aseptics in dental practice and working with other members of the dental team as well as other health professionals [6, 7, 8, 9]. However, the competence in generic skills may be difficult to describe, and generic skills are regularly indicated with other terms, such as soft skills, transferable competencies or key skills [10]. Regardless of the nomenclature, the generic skills in dentistry typically include knowledge analysis, collaboration, communication and problem‐solving skills [11]. Furthermore, the terms may also include elements for utilisation of students’ earlier experiences and knowledge, acting at the interface between theory and practice, giving and getting feedback, developing assessment skills and abilities to summarise tasks, as well as increasing the ability for critical examination of knowledge [12].

In their study, Tuononen et al. [13] stated that graduating students may not consider that the academic competences taught during university studies are the competences that are also needed in working life. Besides, adhering to ethical principles and professional standards is fundamental in dental practice; good communication skills are important for dental practitioners in patient work. In patient work, efficient time management will help the dentist to reduce waiting times and improve practice efficiency. Teamwork and leadership skills are also important for managing a dental practice, and aseptic techniques and risk management are important for maintaining a safe clinical environment and preventing infections. Therefore, increasing students' awareness of the competences should, therefore, be emphasised more already during the undergraduate phase [13]. Additionally, the teachers and supervisors should be aware of the substantial meaning of generic skills in order to enhance their students' understanding of the importance of the matter.

The aim of the study was to evaluate the importance of generic skills in clinical dental practice and how well these skills were achieved during undergraduate dental education.

## 2 Materials and Methods

The Finnish Dental Association gave permission for this study. In Finland, no formal ethical review is required for anonymous and voluntary questionnaire‐based studies [14]. The ethical principles of the Helsinki Declaration were rigorously adhered to in the study, and the contact details of the responsible researcher, as well as information on the aims of the study, were given to the participants. The data contained no identifiers, and the participants could not be identified in the research reports.

The data were based on a national online questionnaire survey called ‘Young Dentist’, which was hosted by the Finnish Dental Association every 3 years since 2011. The ‘Young Dentist’ study includes altogether 39 questions for recently graduated dentists and 10 questions for more experienced colleagues [15]. The questionnaire was sent via an email link to dentists who had received a licence to practise dentistry from the National Supervisory Authority for Welfare and Health (Valvira) during 2018–2020, who had graduated in Finland and who had an email address in the register of the Finnish Dental Association (*n* = 505) [15]. The graduates had received their undergraduate education at the universities providing undergraduate dental education in Finland (Universities of Eastern Finland, Helsinki, Oulu and Turku). The data for the ‘Young dentist’ survey were collected in 2021 between May 18th and June 2nd. Altogether 221/505 recently graduated dentists (response rate 44%) answered the questionnaire.

At the same time (20.5.–1.6.2021), the questionnaire was also sent via an email link for more experienced colleagues, who were collected as a random sample from the member register of the Finnish Dental Association (*n* = 800). A total of 778 of the sample had the email address in the register of the Finnish Dental Association, and 82 of them filled in the questionnaire. The respondents were limited to those who have had recently graduated dentists as their colleagues, and the definite response rate was 80/778 (10%).

The generic skills were selected from the questions from the ‘Young Dentist’ (Table 1). For the recently graduated dentists, the questions were: ‘How important are the (below) mentioned theoretical and clinical skills in the clinical practice of a dentist?’ (1 = not important at all, 2 = slightly significant, 3 = significant, 4 = quite important, 5 = important, 6 = very important) and ‘How did the undergraduate dental education develop the mentioned fields in the clinical practice of a dentist?’ (1 = Very insufficient, 2 = Insufficient, 3 = Quite well, 4 = Well, 5 = Very well, 6 = Excellent).

**TABLE 1:** Skills evaluated in ‘Young dentist’ 2017 and the corresponding ADEE domains.

Generic skills	ADEE domain
Aseptic techniques	I: Professionalism
Communication skills	I: Professionalism
Patient safety and risk management	I: Professionalism
Problem‐solving skills	I: Professionalism
Time management	I: Professionalism
Decision‐making skills	II: Safe and effective clinical practice
Evidence‐based dentistry	II: Safe and effective clinical practice
Financial planning and budgeting	II: Safe and effective clinical practice
Information and communication technology skills	II: Safe and effective clinical practice
Information retrieval skills	II: Safe and effective clinical practice
Knowledge of medical legislation	III: Patient‐centred care
Theoretical knowledge in dentistry	III: Patient‐centred care
Knowledge of social and health care system	IV: Dentistry in society
Management skills	IV: Dentistry in society
Medical ethics	IV: Dentistry in society
Patient encounter skills	IV: Dentistry in society
Sustainable development	IV: Dentistry in society

For the more experienced colleagues, the questions were: ‘How important are the (below) mentioned theoretical and clinical skills in the clinical practice of a dentist?’ (1 = not important at all, 2 = slightly significant, 3 = significant, 4 = quite important, 5 = important, 6 = very important) and ‘What is the level of the theoretical and clinical skills of the young dentists in the (below) mentioned fields?’ (1 = Very insufficient, 2 = Insufficient, 3 = Appropriate, 4 = Good, 5 = Very good, 6 = Excellent).

The results are presented in Tables and Figures. The Figures were produced by using Microsoft Excel for Microsoft 365 MSO (Version 2405). The differences between the categorical values in generic skills evaluated by recently graduated dentists and more experienced colleagues were evaluated by using chi‐square tests (IBM SPSS Statistics 27). The statistical difference was set as *p* < 0.05.

## 3 Results

**FIGURE 1:**
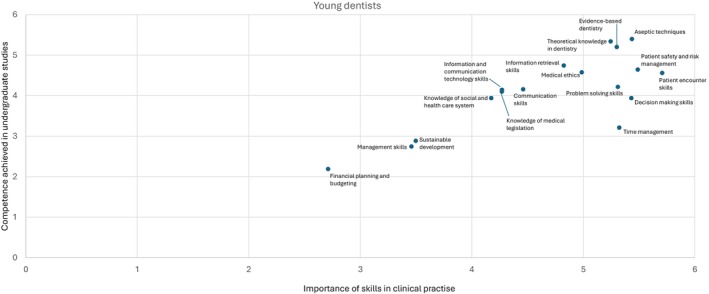
The opinions of recently graduated dentists on the importance of generic skills in clinical dental practice (1 = Not important at all, 2 = Slightly significant, 3 = Significant, 4 = Quite important, 5 = Important, 6 = Very important) and the competence achieved in undergraduate dental education (1 = Very insufficient, 2 = Insufficient, 3 = Appropriate, 4 = Good, 5 = Very good, 6 = Excellent).

Recently graduated dentists stated that patient encounter skills were the most important generic skill in clinical dental practice, and this skill was well achieved during undergraduate studies (Figure 1). Other important skills in clinical dental practice reported by young dentists were aseptic techniques and patient safety and risk management skills, which were also well or very well achieved during undergraduate studies. In contrast, the least important skills in clinical dental practice, and also insufficiently achieved in undergraduate education, were financial planning and budgeting (Figure 1).

**FIGURE 2:**
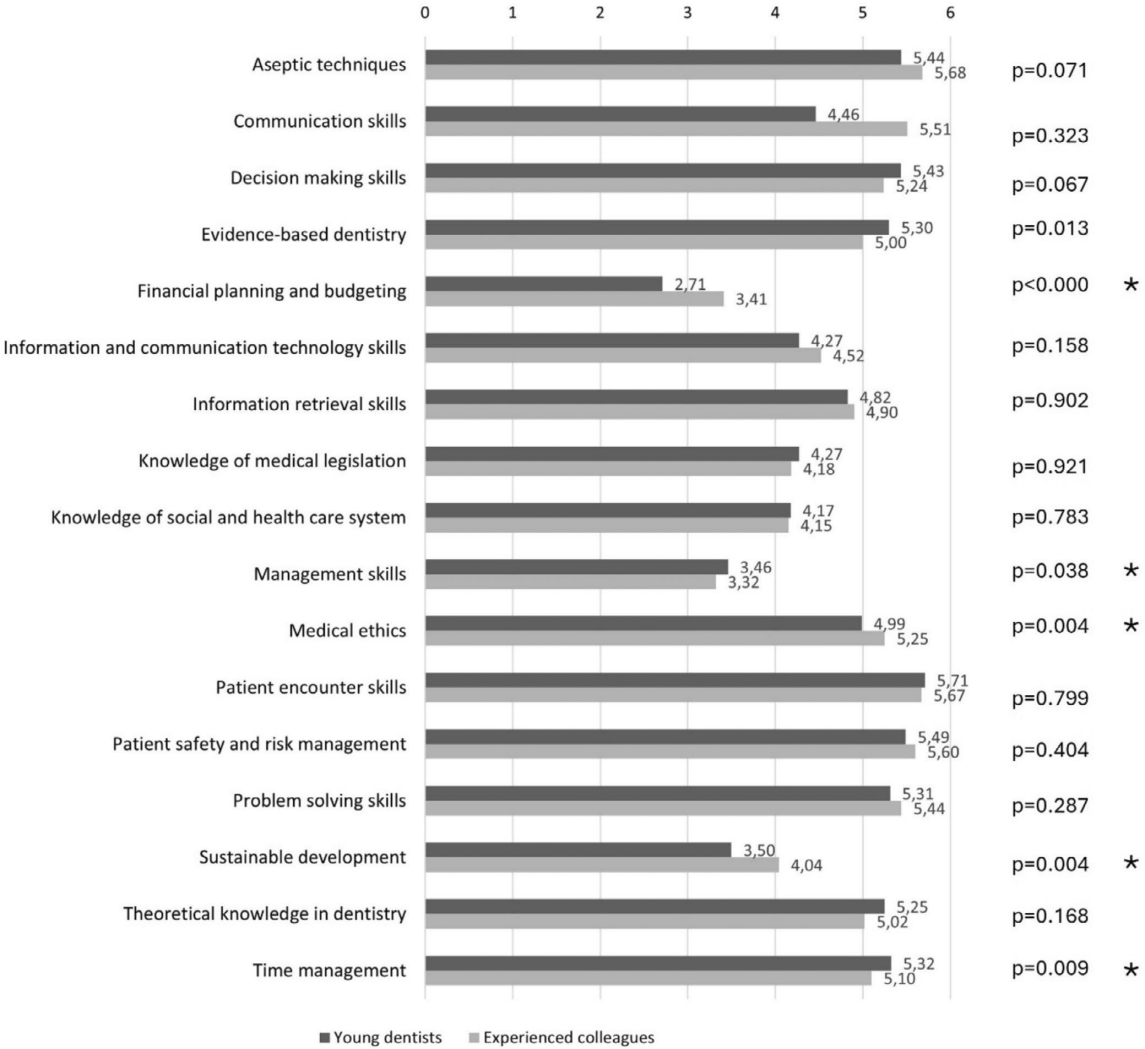
The importance of generic skills in clinical dental practice (mean values) reported by recently graduated dentists and more experienced colleagues. The response options were 1 = Not important at all, 2 = Slightly significant, 3 = Significant, 4 = Quite important, 5 = Important, 6 = Very important.

According to the opinions of more experienced colleagues, the most important generic skills in clinical practice were patient encounter skills, aseptic techniques and communication skills (Figure 2). The management skills were the least important in clinical practice. Significant differences between the opinions of recently graduated dentists and more experienced colleagues concerning the importance of generic skills in clinical practice were in financial planning and budgeting, medical ethics and sustainable development, all these skills in favour of more experienced colleagues. In addition, significant differences in favour of recently graduated dentists were found in management and time management skills.

**FIGURE 3:**
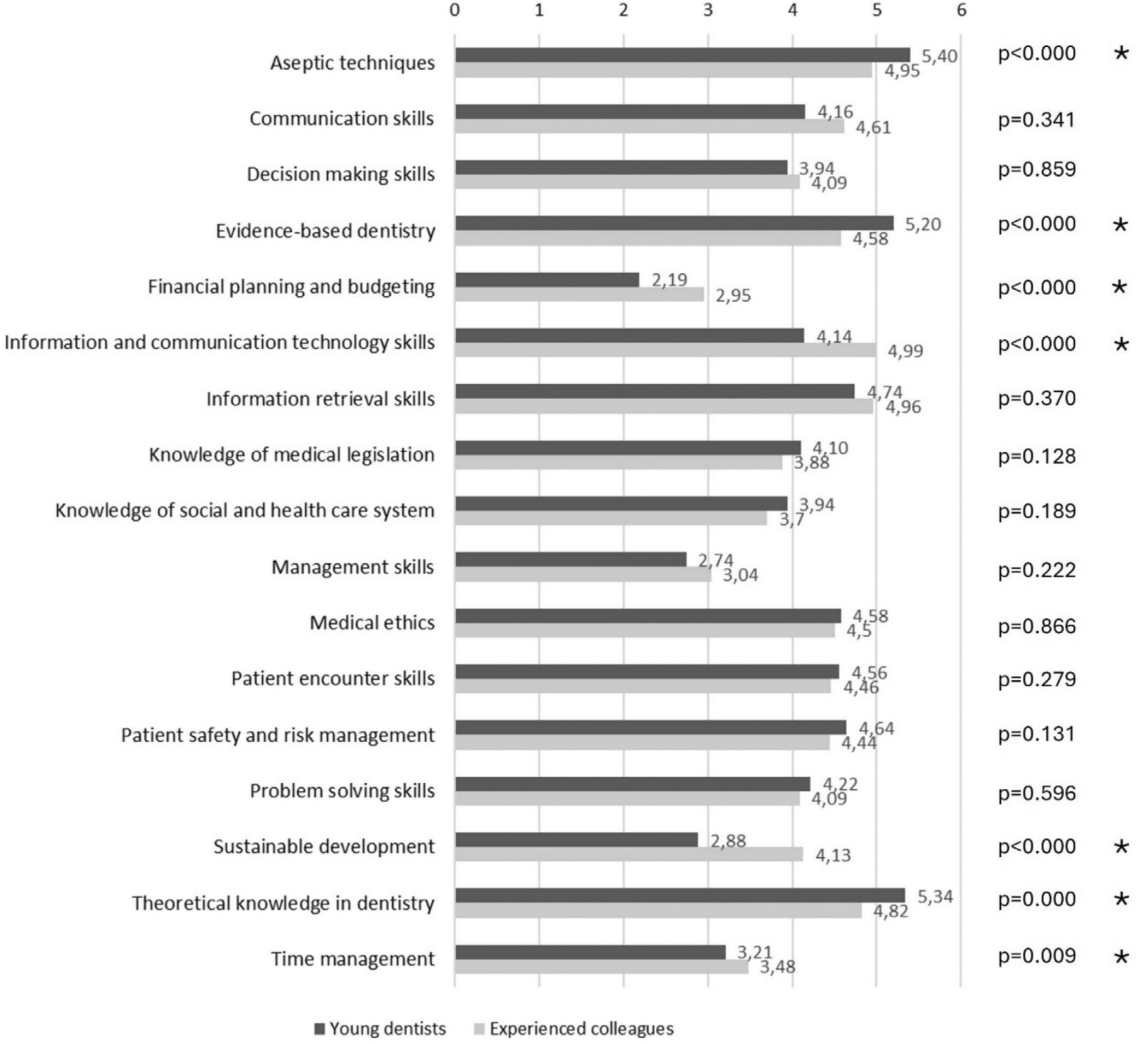
The level of generic skills of young dentists reported by recently graduated dentists and more experienced colleagues. The response options were 1 = Very insufficient, 2 = Insufficient, 3 = Appropriate, 4 = Good, 5 = Very good, 6 = Excellent.

More experienced colleagues reported that skills in information and communication technology, in information retrieval and in aseptic techniques were best achieved among young dentists (Figure 3). The significant differences between the opinions of recently graduated and more experienced colleagues were in skills in information and communication technology, financial planning and budgeting, sustainable development and in time management, all of them on behalf of more experienced colleagues. In addition, significant differences in favour of recently graduated dentists were found in aseptic techniques, evidence‐based medicine and theoretical knowledge in dentistry.

## 4 Discussion

The level of generic skills achieved during undergraduate dental education seems to respond well to the need in clinical dental practice. Both recently graduated dentists and more experienced colleagues shared the same opinion, with minor differences.

Recently graduated dentists valued the generic skills in patient encounter skills as most important in clinical practice, and they reported that those skills were also very well attained in undergraduate studies. Effective communication skills are essential for patient encounters. It is established that focused and clear communication improves patient health and increases patient satisfaction. Interpersonal communication and patient encounter skills are also underlined in ADEE guidelines for dental education [16]. In the present study, the communication skills were reported to be well attained during the education by recently graduated dentists, but interestingly, they did not experience the same for patient encounter skills (Figure 1). The importance of communication skills seems to increase during the years and with professional development: More experienced colleagues found communication skills more important in clinical practice than recently graduated dentists. Both groups did report the patient encounter skills to be the most important generic skill in the dental profession (Figure 2). Fluent communication is the basis of interaction with patients, dental personnel and other health care professionals. This should be developed further in dental undergraduate education by means of harmonising curricula in all countries. In addition, good communication in foreign languages enables dentists to treat the growing number of nonnative language‐speaking patients better and also facilitates the free movement of dental professionals with less challenge in Europe [17, 18].

Aseptic techniques and patient safety and risk management were stated as very important in clinical practice by both recently graduated dentists and more experienced colleagues. The World Health Organisation (WHO) has determined that primary care curricula must include instruction on patient safety (WHO 2021), and patient safety training is part of the ADEE curriculum [2, 19]. Adverse events are reduced through evidence‐based patient safety education [20]. Previous research has found that patient safety training for undergraduate dental students with improved knowledge and interest is effective [21]. It has also previously been found that learning about patient safety is improved by positive attitudes of learners, course satisfaction and improvement of clinical processes [22]. A previous survey of dental students found that students have good qualifications in patient safety, especially in clinical safety skills and communication. Learning related to security risk management was considered the most challenging [23]. It has been shown that dental students have a positive attitude towards teaching and improving patient safety and risk management [24]. Students understand patient safety concepts and techniques to prevent adverse events.

In general, the skills of financial planning and budgeting were not highly valued to be important in clinical practice; on the other hand, they were not well included in undergraduate education. Although economics is more clearly emphasised in the private than in the public sector, the basics of economics should perhaps be more broadly included in the undergraduate curriculum in the future. The same is true for management skills because the dentist is a leader of a team, even if the team only consists of a dental nurse and the dentist herself/himself.

In recent years, interest in environmental sustainability has increased and it has been found to be an important issue also in health care and dentistry [25]. It is recommended that sustainability should be a part of everyday practice, and all dental professionals should be encouraged to take responsibility for it [26]. In the present study, the opinions on the skills concerning sustainable development between recently graduated and experienced colleagues differed noticeably; recently graduated did not find sustainability issues as important as more experienced ones. Moreover, experienced colleagues found skills on the topic attained during the education to be at a good level whereas recently graduated were more doubting. These results point out the significance of embedding sustainable development in educational curricula, also recently reported in the study among dental students in the United Kingdom and recommended by the Association for Dental Education in Europe [27, 28].

The results of the study were based on self‐evaluation, and only minor differences between the self‐evaluations of young dentists and their more experienced colleagues were found. Good self‐assessment skills and ethics should be integral to dental education from the start, ensuring high‐quality patient care and maintaining the reputation and trust of the profession. Dentists often work closely with their assistants, relying heavily on their own skills and self‐assessment abilities. It is crucial for self‐assessment to be objective and precise. It has been shown that during undergraduate dental education, students tend to overestimate their work quality [29]. Gender differences have also been noted, with male students more likely to overestimate their skills [29], especially surgical ones [30]. To improve self‐assessment skills, various methods have been used, including well‐structured forms [31], clear assessment criteria [32], simulated patients [33] and photography [34]. Haptic learning devices also help strengthen self‐assessment without needing more teachers [35]. In addition, over the past years, different learning methods, such as role‐playing, peer‐assisted learning, project work or inquiry‐based learning, have been implemented and evaluated in the teaching of generic skills in undergraduate dental and medical students [36, 37, 38].

The weakness of the study is the low response rate of more experienced colleagues. In addition, the number of years in practice is a significant variable that can influence responses, and it would be interesting to analyse it in further studies. For instance, a dentist with 3–5 years of experience may have a different perspective compared to one with 15 years or more. These limitations may restrain the ability to generalise the results to all levels of experience within the colleagues. The strength of the present study is the study population representing many areas of the country and the reasonably high response rate among recently graduated dentists. Most of the questions have been used in the same format in previous Young Dentist studies conducted four times every fourth year since 2011 to ensure comparability between studies. In addition, inquiring about the same questions from the more experienced colleagues and their assessment of the skills of young dentists brings a valuable addition to the research. Including the perspectives of more experienced colleagues adds significant value to the research by providing an external, practice‐based evaluation of the skills of newly graduated dentists. This validates the self‐assessments of young professionals and offers insights in experienced clinical practice. There were no specific criteria for the assessment, but the opinions used by experienced dentists to assess the skills of recent graduates were based on their clinical experience, ongoing professional education and deep understanding of the dental field, including its core competencies and evolving demands. The evaluations of the more experienced colleagues are based on the years of observing how essential skills—such as communication, decision‐making, technical proficiency and patient management—are applied in daily practice.

The study cannot explicitly describe how the competencies are acquired or assessed their importance in clinical dental practice (e.g., through specific subjects, methods or activities). However, it highlights that generic skills are essential in dentistry. Based on the literature, graduating students often do not recognise the relevance of academic competencies to working life [13]. Therefore, the importance of these skills in clinical practice is inferred through the recommendation to increase students' awareness of them during undergraduate education and to ensure that educators understand the significance of the generic skills in preparing students for professional practice.

In conclusion, according to the opinions of the dentists, undergraduate dental education develops most generic competencies in clinical dental practice; but evolving practices, advancing technologies and health system challenges highlight the growing importance of diverse generic skills in the dental profession.

## Conflicts of Interest

The authors declare no conflicts of interest.

## Data Availability

The data are available from the Finnish Dental Association, but restrictions apply to the availability of these data, which were used under licence for the current study and are, thus, not publicly available. Data are, however, available from the authors upon reasonable request and with permission of the Finnish Dental Association.
